# Alcohol and sudden unexpected death in epilepsy: do not pop the cork

**DOI:** 10.6061/clinics/2020/e1770

**Published:** 2020-04-30

**Authors:** Carla A Scorza, Marcia Guimarães-Marques, Eduardo R C Girão, Mariana Nejm, Josef Finsterer, Manoel J B C Girão, Riad N Younes, Ronaldo R Laranjeira, Fulvio A Scorza

**Affiliations:** I Disciplina de Neurociencia, Escola Paulista de Medicina, Universidade Federal de Sao Paulo (UNIFESP), Sao Paulo, SP, BR; II Krankenanstalt Rudolfstiftung, Messerli Institute. Vienna, AustriaAustria; III Departamento de Ginecologia. Escola Paulista de Medicina, Universidade Federal de Sao Paulo (EPM/UNIFESP), Sao Paulo, SP, BR; IV Centro de Oncologia, Hospital Alemao Oswaldo Cruz, Sao Paulo, SP, BR; V Departamento de Psiquiatria, Escola Paulista de Medicina, Universidade Federal de Sao Paulo (EPM/UNIFESP), Sao Paulo, SP, BR

An article published by Cancer has generated special attention owing to its relevant discussions and future perspectives. In the December issue of this prestigious journal, Zaitsu et al. demonstrated that even moderate alcohol consumption increases the risk of cancer incidence, including gastrointestinal, aerodigestive, breast, and prostate cancers 1,2. To test this hypothesis, the authors evaluated 63,232 volunteers and excluded confounding variables potentially linked to alcohol consumption that might explain alcohol-related cancer risk, including smoking history, high occupational class status, and lifestyle-related comorbidities 1,2. Thus, they clearly demonstrated that individuals who drink a glass of wine or dose of distillate every day for many years have a 5% increased risk of developing cancer 1,2. Remarkably, those who had two drinks every day had a 54% increased risk of developing cancer 1,2. Interestingly, an editorial published simultaneously by Klein et al. from the United States National Cancer Institute showed alarming data regarding the relationship between alcohol consumption and cancer occurrence 2,3. As a conclusion to this article, the authors elucidated the need for the medical community to improve patient education and make their patients aware of the carcinogenic effects of alcohol 2,3.

## Simultaneously, in the field of neurology, why did alcohol consumption by patients attract the attention of epileptologists?

The answer lies in the national case–control study in Sweden assessing whether or not possible specific clinical features are associated with an increased risk of sudden unexpected death in epilepsy (SUDEP) 4. It was clearly demonstrated that individuals with generalized tonic-clonic seizures (GTCS) who sleep alone have a dramatically increased SUDEP risk 4. Among comorbidities, a history of substance abuse or alcohol dependence was associated with a high risk of SUDEP 4. In this line of reasoning, what is the current scenario of SUDEP? Epilepsy is a common, disabling neurological disorder affecting at least 70 million people worldwide 5,6. Epidemiological studies have shown that the prevalence of epilepsy is 6.4 cases per 1,000 people and that the annual incidence is 67.8 cases per 100,000 person-years 5,7. Furthermore, in approximately one-third of the individuals with epilepsy, seizures cannot be sufficiently controlled with the currently available antiepileptic drugs (AEDs) 8-10. In this sense, high seizure frequency and severity are considered major causes of disability, comorbidities, stigma, costs, and mortality 6,8,9. The mortality is highest for those with drug-resistant epilepsy 9. In high-income countries, the standardized mortality ratio ranges from 1.6 to 3.0 while in low and middle-income countries, the corresponding ratio is 19.8 (95% confidence interval: 9.7-45.1) 11-13. Moreover, the standardized mortality ratio is slightly higher in men, children and adolescents, people with epilepsies with a documented etiology, and patients with poor adherence 11.

SUDEP is a leading epilepsy-related cause of death in people with epilepsy 14. SUDEP accounts for 5% to 30% of deaths in individuals with epilepsy, particularly in 20- to 40-year-old patients with chronic, medically refractory epilepsy 15-17. Importantly, a recent practice guideline recommends that clinicians inform parents/guardians of children with epilepsy that SUDEP affects only 1 in 4,500 children per year and 1 in 1,000 adults per year 13,15,18. From the didactic point of view, SUDEP is defined as “the sudden, unexpected, witnessed or unwitnessed, non-traumatic, and non-drowning death in patients with epilepsy, with or without evidence for a seizure, and excluding documented status epilepticus, in which postmortem examination does not reveal a toxicological or anatomical cause for death” 19. Recent research suggests that the most common clinical risk factor for SUDEP is the presence of (nocturnal) seizures, mainly GTCS, and the most effective SUDEP prevention method is good seizure control (reduce seizure frequency to <3/y) 18,20,21. Although the cause(s) of SUDEP are still unknown 22,23, advances in human and experimental research suggest that the mechanism underlying SUDEP is multifactorial, including cardiac arrhythmia, respiratory dysfunction, dysregulation of systemic or cerebral circulation, and seizure-induced hormonal and metabolic changes during and after seizures 20,24. Our understanding of the best way to prevent SUDEP is still incomplete 23. Certainly, seizure control is still the most effective way to manage SUDEP 20,21. Nevertheless, various possible preventive strategies, including reduction of stress, participation in physical activity and sports, dietary management (e.g., omega-3 supplementation), supervision at night, and living with a dog, are promising 25-27.

After reading thus far, readers should be asking about the initial proposal of this editorial, namely the relationship between alcohol and SUDEP. Following this line of reasoning, other potential risk factors for SUDEP that should not be disregarded have also been identified, such as young age at the onset of epilepsy, long duration of epilepsy, dementia, male sex, absence of cerebrovascular disease, asthma, winter climate, and alcohol abuse 28-32. Considering the last additional risk factor, the relationship between alcohol and epilepsy has been linked in medical writings for centuries (since Hippocrates and the Romans), but these complex, multifaceted, and problematic associations have changed over time 32-35. A recent observation is that the prevalence of epilepsy in alcohol-dependent individuals from Western industrialized countries may be at least thrice that in the general population whereas the prevalence of alcoholism is only slightly higher in people with epilepsy than in the general population 35. Clinical data have shown that seizures may occur during alcohol intoxication or the withdrawal period 36,37. Furthermore, it has been consistently demonstrated that individuals with epilepsy who drink moderate or heavy amounts of alcohol could have a high risk of seizures 33. Importantly, alcohol is a risk factor for ischemic cerebral infarction and increases the chances of head trauma, both of which are known factors inducing epilepsy 35,38. In parallel, experimental research has substantially enhanced our understanding of the relationship between alcohol intake and epilepsy 35. More than a decade ago, our research group investigated the effects of alcohol intake and withdrawal on the seizure frequency and brain hippocampal morphology in rats with epilepsy. In brief, we demonstrated that alcohol administrations induced behavioral (frequency of seizures) and neuropathological changes in rats with epilepsy 39. As a main result, we found that the alcohol withdrawal syndrome is crucial for the development of functional and hippocampal abnormalities associated with epilepsy 39. With regard to SUDEP, the scenario becomes even more aggravating. In 2009, Scorza et al. used the pilocarpine model of temporal epilepsy to verify the effects of alcohol consumption on seizure frequency as well as the underlying possible association between alcohol intake and SUDEP occurrence 34,40. The authors showed a significant increase in seizure frequency during the first 2 weeks of alcohol administration, and, quite interestingly, one rat died suddenly after a GTCS, suggesting the presence of an association between alcohol abuse and SUDEP occurrence 34,40. From the clinical point of view, there is a clear suggestion of an association between alcohol abuse and SUDEP, although statistical significance was not seen in studies, making it unclear whether alcohol intake is a risk factor for SUDEP or not 30,34. Along these lines, in a community-based retrospective Finnish case–control study, SUDEP occurred significantly more frequently in men than in women who consumed alcohol regularly and in marked quantities compared to those who consumed alcohol rarely and in small quantities or not at all 34,41,42. In 2017, Lynn et al. investigated the death rate among individuals with epilepsy recorded on the National Drug-Related Deaths Index (NDRDI) 43. The authors performed a descriptive analysis of individuals with a known history of epilepsy in the NDRDI from 2004 to 2013 43. In brief, they demonstrated that a high percentage of individuals with alcohol dependency died from epilepsy and were not under AED therapy at the time of death, highlighting the need for preventive measures for this at-risk group 43. More recently, Chen et al. assessed the frequency and demographic and clinical characteristics of patients with SUDEP in a sudden death cohort (44). For this purpose, the authors verified all out-of-hospital deaths from March 1, 2013, to February 28, 2015, in Wake County, NC, attended by the Emergency Medical Services (44). It was found that SUDEP accounts for approximately 5.3% of the cases of sudden death from any cause among individuals aged between 18 and 64 years (44). Furthermore, mental health disorders and low levels of medication compliance and healthcare utilization were common among patients with SUDEP (44). It was clearly demonstrated that patients with a history of alcohol abuse were more likely to suffer from SUDEP (44).

Overall, from the data presented, are we convinced of the real, complex, and fatal relationship between alcohol consumption and epilepsy? We epileptologists are totally convinced. In addition, further clinical and experimental studies are required to more clearly define the mechanism and obtain accurate epidemiological data on the relationship between alcohol consumption and SUDEP. Finally, it is up to us, as neuroscientists and educators, to inform our students, patients, caregivers, the general population, and the staff in the healthcare system, regarding this issue, which is already a public health problem and not restricted to the field of neurology.

## AUTHOR CONTRIBUTIONS

All authors contributed equally to the manuscript composition.
